# A Study of Phytolith-occluded Carbon Stock in Monopodial Bamboo in China

**DOI:** 10.1038/srep13292

**Published:** 2015-08-25

**Authors:** Jie Yang, Jiasen Wu, Peikun Jiang, Qiufang Xu, Peiping Zhao, Shanqiong He

**Affiliations:** 1 Zhejiang Provincial Key Laboratory of Carbon Cycling in Forest Ecosystems and Carbon Sequestration, Zhejiang A & F University, Lin’an 311300, China; 2 School of Environmental and Resource Sciences, Zhejiang A & F University, Lin’an 311300, China

## Abstract

Bamboo plants have been proven to be rich in phytolith-occluded carbon (PhytOC) and play an important role in reducing atmospheric concentrations of CO_2_. The object of this paper was to obtain more accurate methods for estimation of PhytOC stock in monopodial bamboo because previous studies may have underestimated it. Eight monopodial bamboo species, widely distributed across China, were selected and sampled for this study in their own typical distribution areas. There were differences (*P *< 0.05) both in phytolith content (Phytolith/dry biomass) across leaves, branches and culm, and in PhytOC content (PhytOC/dry biomass) across leaves and branches between species, with a trend of leaf > branch > culm. The average PhytOC stored in aboveground biomass and PhytOC production flux contributed by aboveground biomass varied substantially, and they were 3.28 and 1.57 times corresponding dates in leaves, with the highest in *Phyllostachys glauca McClure* and lowest in *Indocalamus tessellatus* (Munro) Keng f. It can be concluded that it could be more accurate to estimate PhytOC stock or PhytOC production flux by basing on whole aboveground biomass rather than on leaf or leaf litter only. The whole biomass should be collected for more estimation of bamboo PhytOC sequestration capacity in the future.

Increasing greenhouse gas emissions and continuous rising of atmospheric CO_2_ concentrations have become the leading cause of global warming and frequent occurrence of extreme weather events worldwide in recent years^1^,^2^,^3^. According to statistics^4^, as of the end of 2013, global industrial CO_2_ emissions reached 3.6 × 10^10^ t. With further global economic development, the annual CO_2_ emissions will increase further. It is also worth noting that the continuous increase in global average temperature has directly increased soil respiration, accelerating the release of large amounts of carbon from soil^5^,^6^,^7^. Currently, there are two effective ways to control atmospheric CO_2_ content: to reduce emissions and to increase carbon sinks. Studies have shown that soil is the largest carbon pool in terrestrial ecosystems^8^. The total organic carbon content of soil is three times higher than the plant carbon pool^7^,^9^,^10^. Despite the large capacity of the soil to store carbon, most of the organic carbon in soil cannot exist stably for long-term periods because of land use change, complex carbon storage mechanisms and constantly changing environmental conditions^11^,^12^. Hence, it is particularly important to find a safe and effective long-term carbon sequestration mechanism.

Phytolith is a complex silicon-coated carbon compound formed mainly during the growth of *Poaceae, Bambusoideae* and *Cyperaceae* plants. Amorphous silicon in soil is absorbed by these plants and gradually accumulates in plant cells and cell gaps^13^,^14^. PhytOC is the stable organic carbon occluded within amorphous silicon dioxide produced during the formation of phytolith^15^,^16^,^17^. PhytOC enters the soil through the falling and decay of plant parts. In the soil environment, PhytOC remains protected by the outer layer of silicon, which is highly resistant to weathering^18^,^19^ and thus can be sequestered in the soil for a very long time (a few thousand to a few million years). Hence, PhytOC is considered one of the most important mechanisms of long-term terrestrial soil carbon sequestration^20^,^21^. However, due to the different accumulation and distribution of PhytOC in different plants, researchers have not been able to accurately estimate the amount of this component of organic carbon.

*Bambusoideae* plants have been shown to have exceptional capacity for phytolith accumulation^17^. According to statistics^22^,^23^, the total global area of bamboo forests in 2010 was 2.2 × 10^7^ ha. In China, bamboo forests occupied an area of 6.73 × 10^6^ ha in 2010 with the area occupied by *Phyllostachys heterocycla* (Carr.) Mitford cv. *Pubescens* (*Ph. Pubescens*) forests was 4.48 × 10^6^ ha. The total area of other monopodial bamboos was 7.46 × 10^5^ ha. Monopodial bamboo forests accounted for 77.71% of the total area of bamboo forests. In addition, with the development of the bamboo economy, the area of monopodial bamboo forests is projected to increase at a rate of 2.0 × 10^5^ ha per year^23^. Based on this, it was estimated that by 2014, the area of monopodial bamboo forests would be 5.88 × 10^6^ ha, accounting for over 80% of the total area of bamboo forests. The forest carbon sink is one of the most important ways to reduce atmospheric CO_2_^24^,^25^,^26^,^27^.

As part of the forest carbon sink, bamboo forest PhytOC has been studied by a number of scholars in recent years^17^,^22^,^28^,^29^,^30^. Parr *et al.* and Li *et al.* estimated the amount of PhytOC sequestered in bamboo forests worldwide in 2010 and in bamboo forests in China in 2013, respectively^17^,^22^. The estimation by Parr *et al.*^17^ was based on the average of 10 species of sympodial bamboo, and that by Li *et al.*^22^ was based on the average 75 species of bamboo including sympodial and monopodial bamboo. However, their estimates were based on a small collection from small bamboo gardens and based on leaf litter only. Phytolith is formed in different plant organs, and it has been shown that the organic carbon contents in the phytolith extracted from bamboo branches and culms are far higher than those in bamboo leaves^31^. In addition, the PhytOC stock in a single bamboo species across China or worldwide is determined by the combination of the PhytOC content, biomass and total area occupied by the species^22^,^30^,^32^. Further, the total biomass of bamboo branches and culms is much higher than that of bamboo leaves^33^. Hence, we believe that a large amount of PhytOC sequestered in the branches and culms of the bamboo forest ecosystem has been overlooked in previous studies, and the PhytOC stock in bamboo forests, estimated using bamboo leaves, has been severely underestimated. In the current study, eight typical monopodial bamboo species with relatively wide distributions in China were selected. The PhytOC contents and biomass of these species were determined in order to estimate the PhytOC sequestration capacity of monopodial bamboo forests in China and worldwide, and to provide a scientific basis for research on the PhytOC sink in the bamboo forest ecosystem.

## Results

**Figure 1:**
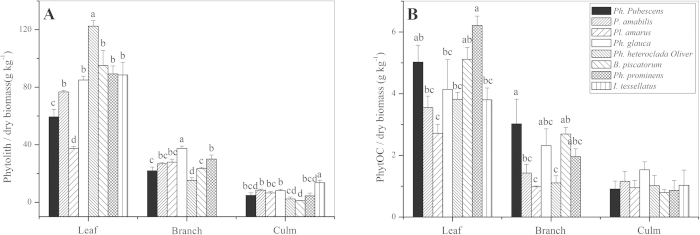
Comparison of Phytolith/dry biomass (**A**), and PhytOC/dry biomass (**B**) in different organs of 8 widely distributed bamboo species. Error bars are standard error (*n* = 4); different lowercase letters indicate significant differences among the bamboo species at *P* = 0.05 level based on the least significant difference (LSD) test.

**Figure 2:**
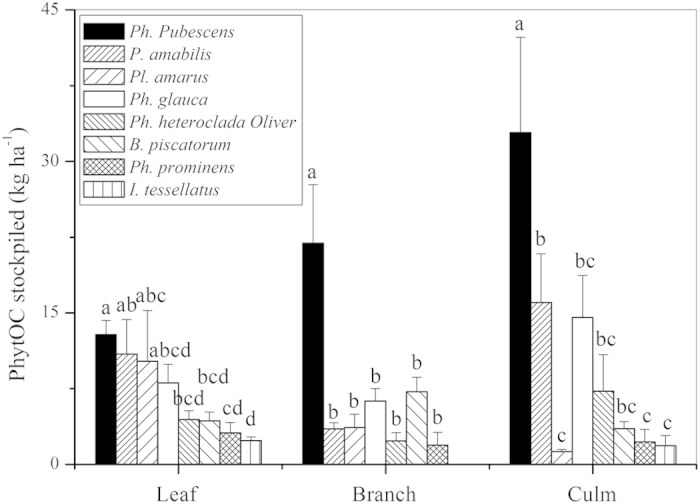
Comparison of PhytOC stockpiled in different organs of 8 widely distributed bamboo species. Error bars are standard error (*n *= 4); different lowercase letters indicate significant differences among the bamboo species at *P *= 0.05 level based on the least significant difference (LSD) test.

Both phytolith contents (Phytolith/dry biomass) and PhytOC contents (PhytOC/dry biomass) varied substantially in the leaf, branch and culm samples among the eight monopodial bamboo species (Fig. 1A,B). Differences between many species were statistically significant (*p* < 0.05) except for the PhytOC content in the culm. Both the phytolith and PhytOC contents showed the following trend: leaf > branch > culm. The leaf phytolith content was highest in the triennial *Phyllostachys heteroclada* Oliver (122 ± 4 g kg^−1^), and lowest in the triennial *Pleioblastus amarus* (Keng) Keng f. (37 ± 1 g kg^−1^) (Fig. 1A). The PhytOC content in leaves ranged from 2.7 ± 0.3 to 6.2 ± 0.3 g kg^−1^ with a mean of 4.3 g kg^−1^ (Fig. 1B). The PhytOC stock per ha was highest in the culm compared with the leaf and branch (Fig. 2), except for *Pl. amarus*, *Phyllostachys prominens* and *Indocalamus tessellatus* (Munro) Keng f. However, the amount of PhytOC stock per ha within the same organ varied among species and significant differences were observed between certain species (Fig. 2).

It was found that the average leaf PhytOC stockpile of all eight monopodial bamboo species was 15.3 kg ha^−1^, with the highest PhytOC stockpile being 24.7 kg ha^−1^ for *Phyllostachys glauca McClure*, and the lowest being 5.1 kg ha^−1^ for *I. tessellatus* (Table 1). The average aboveground biomass PhytOC stockpile of all eight species was 50.2 kg ha^−1^, with the highest PhytOC stockpile of 82.4 kg ha^−1^ and the lowest of 8.4 kg ha^−1^ being observed for the same two species, respectively. PhytOC stocks for the remaining 6 species ranged from 36.2 to 70.7 kg ha^−1^. The average aboveground biomass PhytOC stockpile was 3.28 times that in leaves. The most widely distributed species *Ph. Pubescens* stored 15.1 kg ha^−1^and 55.8 kg ha^−1^ in leaves and aboveground biomass, respectively.

**Figure 3:**
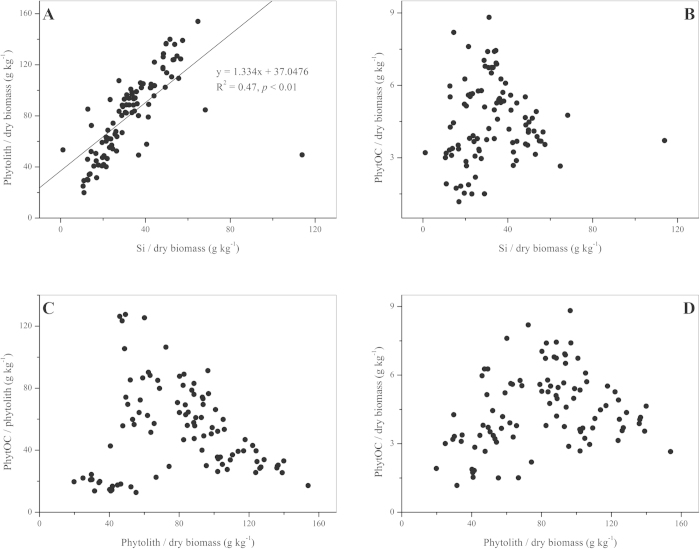
Correlation between 4 parameters for bamboo leaves. (**A**) Si/dry biomass and phytolith/dry biomass, (**B**) Si/dry biomass and PhytOC/dry biomass, (**C**) phytolith/dry biomass and PhytOC/phytolith, (**D**) phytolith/dry biomass and PhytOC/dry biomass.

To determine the relationship between silicon content (Si/dry biomass), phytolith content, and C content in phytolith (PhytOC/phytolith) and PhytOC content in monopodial bamboo leaves, we performed a statistic analysis based on results for all leaf samples. It was found that there was significant linear correlation between silicon and phytolith content (R^2^ = 0.47, *p* < 0.01, Fig. 3A), but no significant linear correlations between the silicon and PhytOC content (Fig. 3B), between the phytolith content and C content in phytolith (Fig. 3C), or the phytolith and PhytOC content (Fig. 3D).

**Figure 4:**
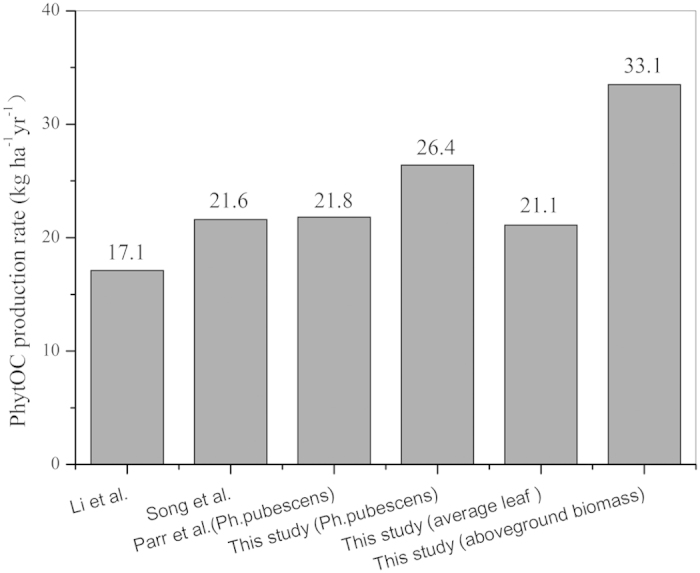
PhytOC production flux as estimated by different researchers. The PhytOC production flux based on leaf litter except for aboveground biamoss.

To compare with the results of previous reports in which the estimation of total PhytOC production flux based on leaf litter (Fig. 4), we conducted an estimation of total PhytOC production flux of monopodial bamboo based both on leaf and aboveground biomass (Table 1). The average PhytOC production flux contributed by leaf and by above biomass was 21.1 kg ha^−1^ yr^−1^ and 33.1 kg ha^−1^ yr^−1^, respectively (Fig. 4). The two species with highest and lowest of PhytOC stockpile annually produced corresponding highest and lowest PhytOC production flux (53.1 kg ha^−1^ yr^−1^ vs 8.9 kg ha^−1^ yr^−1^). The PhytOC production flux for *Ph. pubescens* was 26.4 kg ha^−1^ yr^−1^ for leaves and 44.7 kg ha^−1^ yr^−1^ for aboveground biomass, respectively (Table 1). The PhytOC production flux contributed by aboveground biomass was 1.18 to 1.78 times those estimated by leaf samples for all eight species (mean of 1.57 times).

## Discussion

Plants with different morphological types have different abilities to enrich nutrients in the soil^8^. The morphology and phytolith content varied between different plant species that belong to the same family. It was found that the PhytOC contents in the leaf and branch differed significantly between the eight species (*p* < 0.05, Fig. 1B), and the amount of PhytOC stock per ha within the same organ varied among bamboo species and significant differences were observed between certain species (Fig. 2), suggesting that the PhytOC production capacities of different monopodial bamboo species vary substantially, which may be possibly due to differences in both physiological properties and environments^34^,^35^. There were also considerable differences in PhytOC content among different sympodial bamboo. Parr *et al.* reported^17^ that PhytOC content of 10 species of sympodial ranged from 0.24 to 0.52% for dry leaf biomass. The range of phytolith and PhytOC content in leaves in this study was similar to the results of Li *et al.*^22^, although there was no significant linear correlation observed in this study (Fig. 3D). This finding indicates that it is necessary to measure PhytOC content of the target plant directly rather than to calculate from phytolith content. We observed both a positive liner relationship between phytolith content and PhytOC content in Chinese grassland^36^ and a nonsignificant relationship in sympodial bamboo^17^ and in *Triticum aestivum*, *Setaria italica* and *Setaria viridis*^37^. This could be explained by the results showing that the carbon and silicon contents in different forms of phytoliths, which were isolated from different species or from different organs of the same plant, varied substantially^15^. There were many differences in PhytOC stockpile when other plants belonging to the same grass family as bamboo were compared. The PhytOC stock in the aboveground biomass of eight monopodial bamboo species ranged from 8.4 to 82.4 kg ha^−1^, which was considerably higher than that in millet (5.45 kg ha^−1^)^37^, grassland (1.64 to 10.36 kg ha^−1^)^36^, wetland (0.82 to 21 kg ha^−1^)^38^, rice (7.09 to 34.09 kg ha^−1^)^32^ and wheat (1.64 to 10.36 kg ha^−1^)^39^, but lower than that in sugarcane (32.73 to 98.18 kg ha^−1^)^21^. A likely reason may be that in grassland systems, although most grasses belong to the same grass family (*Gramineae*) as bamboo^36^, the total biomass per hectare is relatively low. Rice, millet and wheat also produce a relatively small biomass since they are mainly planted to harvest seeds rather than for production of their culm and leaves. Sugarcane is a type of silicon-loving plant, and a large amount of silicon absorption directly results in an increase in phytolith accumulation. Additionaly, the biomass per hectare of sugarcane is much higher than that of general monopodial bamboo forests^40^. Both high phytolith accumulation and greater biomass of sugarcane resulted in its much greater amount of aboveground PhytOC stock per unit area compared to monopodial bamboo.

The amount of PhytOC sequestered in the bamboo branches and culms has been largely overlooked in earlier studies. Previous researchers estimated the PhytOC production flux in bamboo forest ecosystems based on the PhytOC content in the leaves of a one bamboo species^17^ or on the mean value of several different morphological types^22^. This is because they believed that only the phytolith in leaves can accumulate in the soil through the falling and degradation of plant leaves^17^,^20^,^21^, whereas the branches and culm are not returned to the bamboo forest ecosystems. However, usually a stable number of bamboo branches and culm are always standing in bamboo forest ecosystems, although new bamboo individuals grow to be harvested every other year^33^. Therefore, the amount of CO_2_ sequestered as PhytOC in bamboo leaves, branches and culm is stable over time, either in bamboo ecosystems or in harvested bamboo timber, because PhytOC is thought to be a stable organic carbon. The PhytOC stock of leaves accounted for 19.7% to 60.4% of the aboveground PhytOC stock, with an average of 30.1% and 27.1% for the widely distributed *Ph*. *Pubescens* bamboo (Table 1). When PhytOC stock is expressed as PhytOC production flux, the difference between leaf and aboveground biomass decreased (Table 1), with leaves annually contributing 56.3 to 84.5% of total PhytOC with an average of 63.7%. However, the average aboveground PhytOC flux of all eight monopodial bamboo species (33.1 kg ha^−1^ yr^−1^) was much more than that of leaves (21.1 kg ha^−1^ yr^−1^). The value of leaf PhytOC flux in this study was generally similar to previous reports (Fig. 4), e.g. the average leaf PhytOC flux was similar to that by Song *et al.*^28^ and higher than that by Li *et al.*^22^ which were both based on average leaf of different species, while leaf PhytOC flux (26.4 kg ha^−1^ yr^−1^) for *Ph*. *Pubescens* this study was somewhat higher than that (21.8 kg ha^−1^ yr^−1^) by Parr *et al.*^17^ (Fig. 4). It can be concluded that it could be more accurate to estimate PhytOC stock or PhytOC production flux by basing on whole above ground biomass than on leaf or leaf litter only. Of cause, it will be much more accurate if estimation of PhytOC stock or PhytOC production flux based on the whole bamboo biomass including below- and above-ground biomass.

## Materials and Methods

### Experimental site

In this study, eight bamboo species, i.e., *Ph. glauca*, *Ph. prominens*, *Pl. amarus*, *Bambusa piscatorum* McClure, *Ph. heteroclada* Oliver, *Pseudosasa amabilis* (McClure) Keng f., *I. tessellatus* and *Ph*. *Pubescens*, were selected for sampling in Zhejiang and Anhui Provinces (Table 2). For each species, samples were collected from four plots, which were used as four replicates. Standard plots and standard sample plants were selected. We select one individual bamboo of each age to determine silicon content, phytolith content, C concentration in Phytolith, PhytOC content and calculate biomass of each organ. All leaves, branches and culms of each sample plant were weighed separately, and for each organ, 500-g samples were transported to the laboratory. Each sample was washed three times with ultra-pure water, dried at 70–75 °C, weighed and biomass per unit area was calculated. The sample was then ground and mixed thoroughly for further testing.

### Sample measurements

Microwave digestion^1^,^14^ was used for phytolith extraction. After extraction, a potassium dichromate solution (0.8000 M) was used to detect whether the organic matter surrounding the phytolith had been completely removed^17^. The extracted phytolith was subjected to centrifugation, washing, and drying at 65 °C for 24 h before been weighed. The phytolith content was then calculated. The PhytOC was determined using the PhytOC alkali spectrophotometry method^41^. The molten lithium metaborate-spectrophotometry method^42^ was used to determine the Si and P contents in the samples. In parallel with sample testing, standard soil (GBW07405) and plant (GBW07602) samples were also tested to verify the accuracy of the measurements.

### Data processing and analysis

Statistical analysis was performed using Excel worksheets. The data were analyzed by ANOVA, and means were compared with the LSD test using the SPSS software (SPSS 13.0 for windows, SPSS Inc., Chicago, USA). Origin 8.5 was used to plot the figures. The related formulas are as follows:


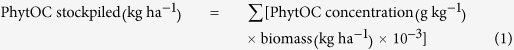



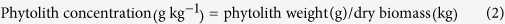



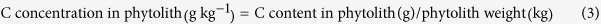



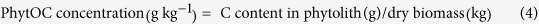



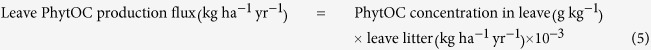


The leave PhytOC production flux was calculated by reference the ratio of living leave biomass to annual litter amount^30^,^31^,^40^.


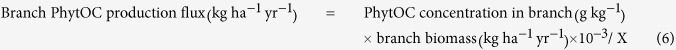



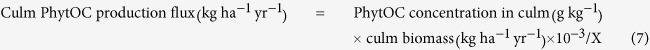


X: The ages of different bamboo species

Biomass PhytOC production flux (kg ha^−1^ yr^−1^) = (5) + (6) + (7)

## Additional Information

**How to cite this article**: Yang, J. *et al.* A Study of Phytolith-occluded Carbon Stock in Monopodial Bamboo in China. *Sci. Rep.* **5**, 13292; doi: 10.1038/srep13292 (2015).

## Figures and Tables

**Table 1:** PhytOC stockplie and PhytOC production flux of different Monopodial bamboo in China.

**Bamboo species**	**PhytOC stockpiled in leaf (kg ha**^**−1**^**)/ percentage to biomass(%)**	**Total PhytOC stockpiled in aboveground biomass (kg ha**^**−1**^)	**PhytOC production flux of leaf (kg ha**^**−1**^**yr**^**−1**^**)/ percentage to biomass(%)**	**PhytOC production flux of aboveground biomass (kg ha**^**−1**^**yr**^**−1**^)
*Ph. glauca*	24.7/30.0	82.4	33.2/62.5	53.1
*Ph. Prominens*	17.1/47.2	36.2	22.3/84.5	26.4
*Pl. amarus*	13.9/19.7	70.7	18.8/62.7	30.0
*B. piscatorum*	12.7/28.2	45.1	16.9/61.2	27.6
*Ph. heteroclada Oliver*	13.0/31.0	42.0	17.6/64.9	27.1
*P. amabilis*	20.8/34.1	61.0	26.4/56.3	46.9
*I. tessellatus*	5.1/60.7	8.4	7.1/79.8	8.9
*Ph. Pubescens*	15.1/27.1	55.8	26.4/59.1	44.7
Average	15.3/30.5	50.2	21.1 /63.7	33.1

**Table 2:** Characteristics of the sampling sites.

**Bamboo species**	**Distribution**	**Distribution area (10**^4^ **ha)**	**Sample site coordinates**		**Rock type**	**Bamboo density (n**/**ha)**^**✶**^
			**N**	**E**		
*Ph. Pubescens*	Anhui Jiangxi Zhejiang	504.23	30°14'22″	119°2'30″	Tufa	2.2 × 10^3^
*Pl. amarus*	Zhejiang Hubei	1.12	30°11'31″	119°51'1.1″	Tufa	7.8 × 10^4^
*Ph. glauca*	Zhejiang Anhui	4.57	30°29'24″	119°9'1.2″	Standstone	2.8 × 10^4^
*Ph. prominens*	Zhejiang	0.59	29°48'0.1″	119°34'24″	Tufa	9.7 × 10^3^
*P. amabilis*	Zhejiang Jiangxi Hainan	4.28	30°15'43″	119°43'38″	Standstone	3.7 × 10^4^
*B. piscatorum*	Zhejiang Sichuan	2.21	30°19'5.9″	119°27'20″	Tufa	1.5 × 10^5^
*Ph. heteroclada* Oliver	Zhejiang Hubei	2.94	30°18'55″	119°27'8.6″	Tufa	5.3 × 10^4^
*I. tessellatus*	Zhejiang Anhui Fujian	5.52	30°20'36″	119°26'18″	Tufa	6.1 × 10^4^

^✶^n means the number of bamboos.
